# Laboratory evaluation of the Sigma Transwab® transport system for the recovery of *Candida* species using the Clinical and Laboratory Standards Institute (CLSI) document M40-A2

**DOI:** 10.1007/s10096-020-04062-9

**Published:** 2020-10-13

**Authors:** E. Elcocks, E.C. Adukwu

**Affiliations:** Centre for Research in Biosciences, Faculty of Health and Applied Sciences, University of the West of England, Bristol, BS16 1QY UK

**Keywords:** Swabs, *Candida*, *C. auris*, Sigma Transwab®

## Abstract

Rising healthcare complications due to fungal infections increase the importance of efficient specimen collection and maintenance systems for correct identification and diagnosis. The CLSI M40-A2 protocol provides guidelines for laboratories assessing quality of medical transport devices, including swab transport systems (STS). This study assessed the efficiency of the Sigma Transwab® foam and flock swab in recovering and maintaining viability of different *Candida* spp. including *C. auris*, in different test conditions. Both swab types recovered and maintained viability of all *Candida* spp. with greater CFU at room temperature after incubation (24 and 48 h) in comparison with swabs maintained at 4 °C.

## Introduction

Invasive fungal diseases are a major global health problem with mortality rates between 30 and 90% and a large proportion of these diseases caused by of *Aspergillus*, *Cryptococcus*, *Candida*, or *Pneumocystis* [1]. *Candida* spp. have been identified as a leading cause of pathogenic diseases, with candidiasis known to affect more than a quarter of a million patients worldwide annually [2]. This increase in opportunistic fungal pathogens can be linked to several factors including chemotherapy, transplant situations which result in an increase in immunocompromised patients [3]. One species of *Candida* that is of particular concern is *C. auris.* Since the first report of *C. auris* in 2009 [4], there have been concerns of rapid spread across the globe [5]; misidentification with other *Candida* spp. [6–9]; seriousness of associated infections including reports of isolation from the bloodstream, urinary tract, ear canal, wounds, heart muscle and bone [10]; high mortality rate, related to bloodstream infections [10–12]; and its antifungal resistance [6, 13].

Rapid and efficient isolation and identification of infectious agents are paramount to successful treatment. Medical transport devices, particularly swab transport systems (STS), have been used in clinical and laboratory diagnosis and are often used for their low cost, ease of use and ability to maintain microorganism viability over extended periods of time [14]. The Clinical and Laboratory Standards Institute (CLSI) M40-A2 is an approved standard which outlines testing procedures for liquid transport systems and provides manufacturers and end-users with a criteria for compliance [15]. This standard focuses on transport and collection devices and recovery of specimen, focuses largely on bacteria and only identifies yeasts for quality control of urine transport systems. Thus, it is important to assess and compare efficiency and effectiveness of swabs and collection devices in maintaining and recovering the integrity of pathogenic yeast specimen. In the study by Gizzie and Adukwu [14], the authors showed that both flock and foam Sigma Transwab®, swab types, were efficient at recovering and maintaining the recommended organisms in the M40-A2 standard including the difficult pathogen *Neisseria gonorrhoea.* The focus of this current study was to assess and compare these two swab types, Sigma Transwab® foam and flocked swab, for maintenance of viability and recovery of different *Candida* spp., including *C. auri*s in vitro. This investigation is important as transport, maintenance and recovery of clinically important fungal pathogens are of relevance to medical and clinical laboratories and to ensure accuracy of diagnosis.

## Methods

The CLSI M40-A2 protocol was used with some minor adaptations. The STS used in this study were both manufactured by Medical Wire and Equipment (MWE; Corsham, UK) and included their Sigma Transwab® foam (MW176S) and Sigma Transwab® Purflock® (MW176PF); both swab types are recommended for use for wound, skin and throat and utilise a liquid amies–carrying media. The MW176S employs a cellular foam bud, while the MW176PF employs a flocked fibre tip. *Candida* spp. used in this study include *C. auris* NCPF 8971, *C. albicans* NCPF 3179, *C. tropicalis* NCPF 3111, *C. parapsilosis* ATCC 22019 and *C. glabrata* ATCC 2001. Smears on microscope slides were produced for each species of *Candida*, and a Gramme stain was performed*. Candida* spp. stored on microbank beads at − 80 °C were grown on Sabouraud Dextrose Agar (SDA; EO Labs, Bonnybridge, UK) at 30 °C for 48 h and used to inoculate 0.85% physiological saline (OXOID, Basingstoke, UK) until turbidity reached McFarland Standard 0.5 (OD_625nm_ 0.08–0.1) with a final working dilutions of 10^4^ and 10^3^ CFU/ml, with 100 μl aliquots dispensed in triplicates into a 96-well plate. The swabs were then immersed in the aliquots, and the dilutions were absorbed for a period of for 10 s after which the swabs were placed in the transport medium and maintained at either room temperature (RT) or 4 °C for 0, 24 and 48 h (T0/T24/T48). The swabs were removed from each well containing the swab/aliquot, rolled on the agar medium (SDA agar) and incubated at 30 °C for 48 h according to the CLSI M40-A2 standard. Following the incubation period, the colonies were enumerated and CFU values determined. The M40-A2 standard indicates that using the roll plate method, the enumerated counts should be ≥ 5 CFU after the specified holding period for specimen held at RT or at 4 °C, when the dilution is compared with the counts from T0 for the STS to be acceptable.

## Results

**Fig. 1:**
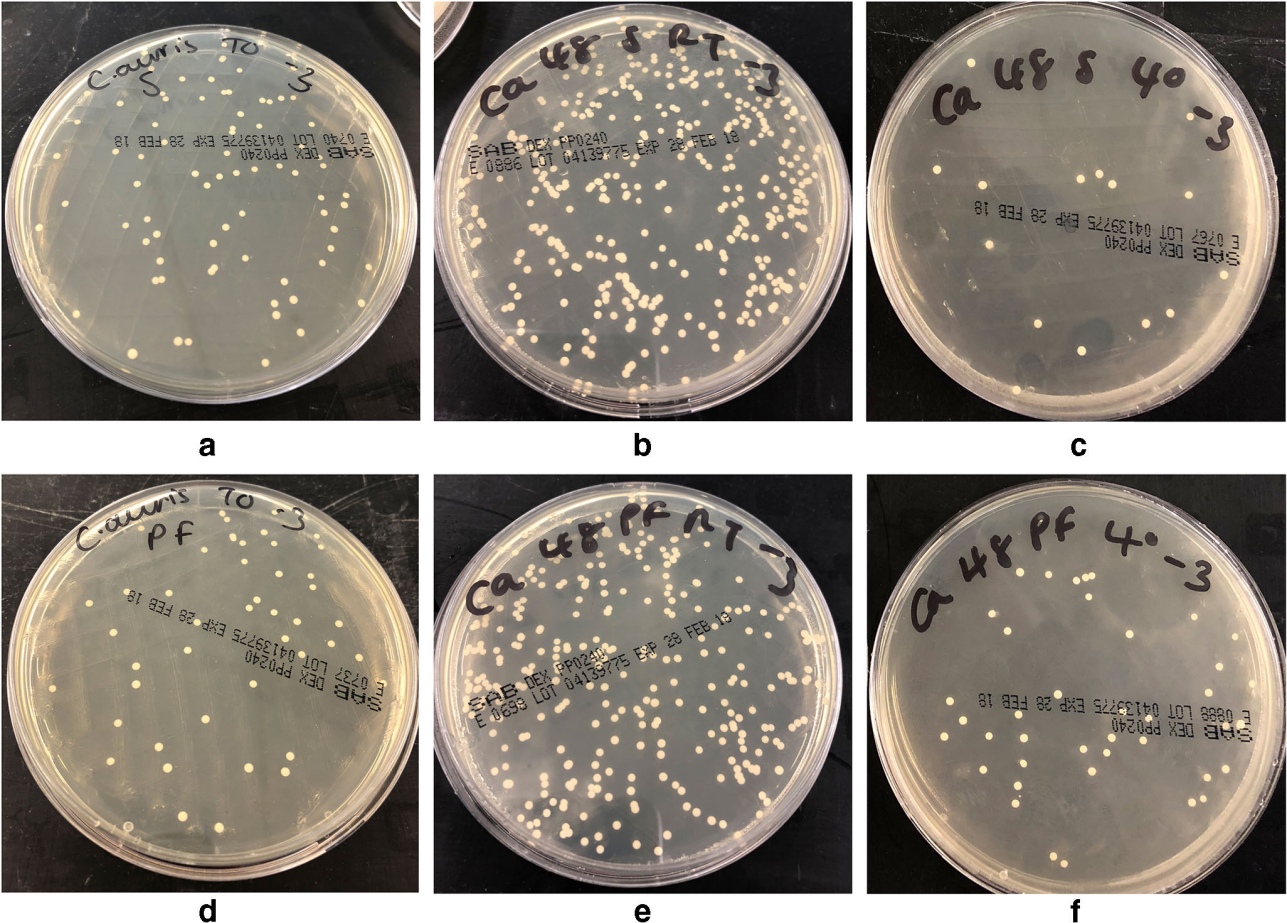
Enumeration of *Candida auris* on Sabouraud dextrose agar plates following maintenance at **a** Sigma Transwab® foam at T0; **b** Sigma Transwab® foam at T48 and RT; **c** Sigma Transwab® foam at T48 and 4 °C; **d** Sigma Transwab® Purflock® at T0; **e** Sigma Transwab® Purflock® at T48 and RT; and **f** Sigma Transwab® Purflock® at T48 and 4 °C

Results from Gram staining procedure showed that all *Candida* spp. presented the Gram-positive appearance with typical round/oval budding morphology. There were no differences observed with the swab types in the shape and morphology of the test microorganism; however, we noticed that the *C. tropicalis* NCPF 3111 comprised budding yeast cells with some hyphal germ tubes and pseudohyphae. All strains of *Candida*, including *C. auris*, were successfully recovered by both foam, flocked swabs at room temperature and at 4 °C, and were found to be complaint with the M40-A2 criteria (Table 1). At time 0, there was no significant difference between the CFU between the *Candida* spp. recovered using both flock and foam swabs at room temperature and at 4 °C; however, after incubation for 24 h and 48 h, the CFU of the test organisms at room temperature was consistently greater than those grown at 4 °C (Fig. 1).

**Table 1:** Qualitative results of bacterial recovery of *Candida* spp. isolated from the Sigma Transwab® foam (MW176S) and Sigma Transwab® Purflock® (MW176PF) at room temperature and at 4 °C. Values are expressed as mean CFU of three replicates (*n* = 3)

Organism	Swab	Temp.	Dilution 10^−2^				Dilution 10^−3^			
			0 h	24 h	48 h	Compliance	0 h	24 h	48 h	Compliance
*Candida auris* NCPF 8971	Foam	RT	TNTC	TNTC	TNTC	✓	51	50	TNTC	✓
	Foam	4 °C	TNTC	179	183	✓	51	16	20	✓
	Purflock	RT	TNTC	TNTC	TNTC	✓	35	55	TNTC	✓
	Purflock	4 °C	TNTC	TNTC	208	✓	35	43	29	✓
*Candida albicans* NCPF 3179	Foam	RT	164	TNTC	TNTC	✓	14	117	255	✓
	Foam	4 °C	164	153	134	✓	14	14	13	✓
	Purflock	RT	176	TNTC	TNTC	✓	14	92	200	✓
	Purflock	4 °C	176	100	133	✓	14	12	17	✓
*Candida tropicalis* NCPF 3111	Foam	RT	128	TNTC	TNTC	✓	14	143	TNTC	✓
	Foam	4 °C	128	71	66	✓	14	4	5	✓
	Purflock	RT	102	TNTC	TNTC	✓	12	67	146	✓
	Purflock	4 °C	102	77	55	✓	12	5	9	✓
*Candida parapsilosis* ATCC 22019	Foam	RT	158	TNTC	TNTC	✓	7	46	236	✓
	Foam	4 °C	158	58	109	✓	7	10	6	✓
	Purflock	RT	135	TNTC	TNTC	✓	13	46	121	✓
	Purflock	4 °C	135	120	154	✓	13	9	13	✓
*Candida glabrata* ATCC 2001	Foam	RT	198	96	269	✓	16	8	20	✓
	Foam	4 °C	198	71	113	✓	16	4	10	✓
	Purflock	RT	171	182	260	✓	18	21	36	✓
	Purflock	4 °C	171	197	165	✓	18	17	23	✓

*TNTC*, too numerous to count; *RT*, room temperature

## Discussion

Swab transport systems have been used in the maintenance and management of specimen. In clinical settings, these STSs aid accuracy and timeliness of diagnosis, which highlights their importance. The type and quality of swab is also important in enabling efficient management of specimen, hence the development of CLSI M40-A2 standard. Following the protocols set out by this standard, we investigated and compared the recovery of *Candida* spp. using the foam and flocked Sigma Transwab® swabs. The STS used in this study efficiently recovered and maintained the growth of *Candida* spp. including the yeast *C. auris*, which is of particular interest to clinicians due to misdiagnosis, link to nosocomial infections and drug resistance of this pathogen. While the recent study by Scansen et al. [16] and Gandhi et al. [17] demonstrated recovery of several *Candida* spp. and other pathogenic fungi using other commercial STS based on the M40-A2 protocol, they did not investigate recovery of *C. auris*. This study investigates the recovery and maintenance of different *Candida* spp. including *C. auris* using the M40-A2 protocol. The M40-A2 does not currently directly address the recovery of yeasts, with the exception of guidance for urine transport systems [15]. In this study, we also found that when using the protocol for adjusting initial inocula, enumeration of the yeasts was lower than that typically determined from bacteria cells, which is possibly as a result of the characteristic differences in sizes of bacteria and yeast; bacteria are smaller in size 0.2 to 2.0 μm in diameter and 2 to 8 μm in length, while the yeasts range between 2 and 60 μm [18, 19].

Of the *Candida* spp. used within this study, only *C. auris* is a known clinical isolate, and in future studies, we recommend using a panel of clinically pathogens which would better reflect the use of STS in clinical situations. However, using the reference strain cultures is in line with the M40-A2 protocol, which utilises quality control strains for testing STS. While the study by Scansen et al. [16] suggested that foam swabs were superior to flocked swabs particularly when used in antigen-testing experiments, in our study, we did not notice any significant differences in the recovery of the *Candida* spp. using both swab types. This study provides evidence of recovery of relevant yeasts including organisms of clinical importance such as the *C. albicans* and *C. auris* using the MWE Sigma Transwab® foam and flock swabs and offers clinical laboratories confidence to utilise these swabs in maintenance and transport of pathogenic and non-pathogenic yeasts.
